# Statistical Evaluation of a Fully Automated Mammographic Breast Density Algorithm

**DOI:** 10.1155/2013/651091

**Published:** 2013-05-08

**Authors:** Mohamed Abdolell, Kaitlyn Tsuruda, Gerry Schaller, Judy Caines

**Affiliations:** ^1^Department of Diagnostic Radiology, Dalhousie University, Halifax, NS, Canada B3H 2Y9; ^2^Department of Diagnostic Imaging, Capital District Health Authority, Halifax, NS, Canada B3H 2Y9; ^3^Division of Medical Education/Informatics, Dalhousie University, Halifax, NS, Canada B3H 2Y9; ^4^Nova Scotia Breast Screening Program, Halifax, NS, Canada B3H 2Y9

## Abstract

Visual assessments of mammographic breast density by radiologists are used in clinical practice; however, these assessments have shown weaker associations with breast cancer risk than area-based, quantitative methods. The purpose of this study is to present a statistical evaluation of a fully automated, area-based mammographic density measurement algorithm. Five radiologists estimated density in 5% increments for 138 “For Presentation” single MLO views; the median of the radiologists' estimates was used as the reference standard. Agreement amongst radiologists was excellent, ICC = 0.884, 95% CI (0.854, 0.910). Similarly, the agreement between the algorithm and the reference standard was excellent, ICC = 0.862, falling within the 95% CI of the radiologists' estimates. The Bland-Altman plot showed that the reference standard was slightly positively biased (+1.86%) compared to the algorithm-generated densities. A scatter plot showed that the algorithm moderately overestimated low densities and underestimated high densities. A box plot showed that 95% of the algorithm-generated assessments fell within one BI-RADS category of the reference standard. This study demonstrates the effective use of several statistical techniques that collectively produce a comprehensive evaluation of the algorithm and its potential to provide mammographic density measures that can be used to inform clinical practice.

## 1. Introduction

 Breast density refers to fibroglandular tissue in the breast and is one of the top major risk factors for breast cancer. Women with extremely dense breasts (75% or greater mammographic density) have a four- to sixfold increase in the risk of developing breast cancer compared to those with fatty breasts (less than 25% density) [1–3]. 

Traditionally, visual assessment by radiologists has been used to characterize and quantify mammographic density (and a woman's risk for breast cancer) using Wolfe Grades, Tabar Patterns, Boyd Scales, or the American College of Radiologists' (ACR) Breast Imaging Reporting and Data System (BI-RADS) density lexicon [4–7]. Despite good reproducibility, methods used to characterize mammographic density have shown weaker associations with breast cancer risk compared to methods quantifying mammographic density [2, 3, 8] and suffer from inter- and intraobserver variability.

The ACR has stated that radiologists' visual assessments of percent breast density using the BI-RADS lexicon are “not reliably reproducible” [9]. This fundamental lack of reproducibility has led to the development of various semi- and fully automated algorithms to quantify percent breast density as a means to overcome inter- and intraobserver variability. It is therefore important to apply rigorous statistical methods to evaluate the performance of these algorithms.

### 1.1. State of the Art

Area-based methods used to quantify mammographic density have produced reliable and standardized mammographic density measurements on a continuous scale. The de facto standard of such methods is the Cumulus software [10, 11]. Using Cumulus, a digitized film-screen mammogram is displayed and a trained user selects a threshold value to separate the breast area from the background (i.e., the region of interest). A second threshold is then selected to identify regions of dense breast tissue, and the percent breast density is calculated as the area of dense tissue divided by the area of the region of interest. Despite being a proven predictor of breast cancer risk, the semiautomated nature of Cumulus' breast density assessments is susceptible to inter- and intraobserver variability and could be improved by a fully automated method. Additionally, this software is intended for use with digitized film-screen mammograms. As 90% of certified mammography units in the USA are now full-field digital [12], a software for use with full-field digital mammograms (FFDMs) is needed.

Volume-based methods theoretically yield accurate estimates of mammographic density and so it is simply assumed that volume-based density estimates are associated with breast cancer risk, as has been demonstrated to be the case for area-based estimates (both visually and algorithmically assessed) [13, 14]. Volumetric methods use “For Processing” FFDMs and DICOM header information to calculate density. Yet, volume-based estimates have not been shown to demonstrate a similarly strong association with breast cancer risk [11, 15, 16]. Additionally, the underlying distribution of mammographic density estimates from volumetric methods is significantly more left-skewed than that of area-based methods (typical range 0–40% versus 0–100%) [17], making them difficult to interpret by radiologists, who are not simply able to visualize mammographic density as a volumetric construct [11, 15].

The assessment of the agreement between percent breast density algorithms and an expert radiologist should necessarily quantify the consistency or reproducibility of measurements made by these two “raters” on the same set of digital mammograms. The intraclass correlation coefficient (ICC) provides such a measure of agreement [18]. The Bland-Altman plot is another way to assess agreement between raters. Scatter and box plots can also yield insights into the level of agreement between raters. Yet, much of the literature validating emerging density measurement algorithms relies on the use of the Pearson correlation coefficient, *ρ*, which is a measure of the linear dependence between two raters and can be quite high despite the agreement being poor [18, 19]. Overall percent agreement is another statistic that is used to assess agreement but is also flawed as it does not factor in any inherent inter- and intrarater variability [19]. Reporting of a single numerical measure of agreement alone is one-dimensional and does not present a comprehensive perspective on algorithm performance.

This paper presents several statistical methods that collectively provide a more comprehensive evaluation of the performance of a fully automated area-based image analysis algorithm that generates percent breast density measures from FFDMs.

## 2. Materials and Methods

138 “For Presentation” FFDMs collected from the Capital District Health Authority in Nova Scotia were retrospectively analyzed. Images were acquired on Siemens full-field digital mammography machines and automatically postprocessed by the manufacture's proprietary software at the time of acquisition. This early stage work has focused on the mediolateral oblique views and excluded craniocaudal views as it has been shown in the literature that mammographic density estimates from only one view are sufficient to indicate breast cancer risk [20]. In addition, the ACRs' National Mammography Database breast density element definition stipulates that “if left and right breasts differ, use the higher density” [21].

### 2.1. Percent Density Analysis

**Figure 1:**
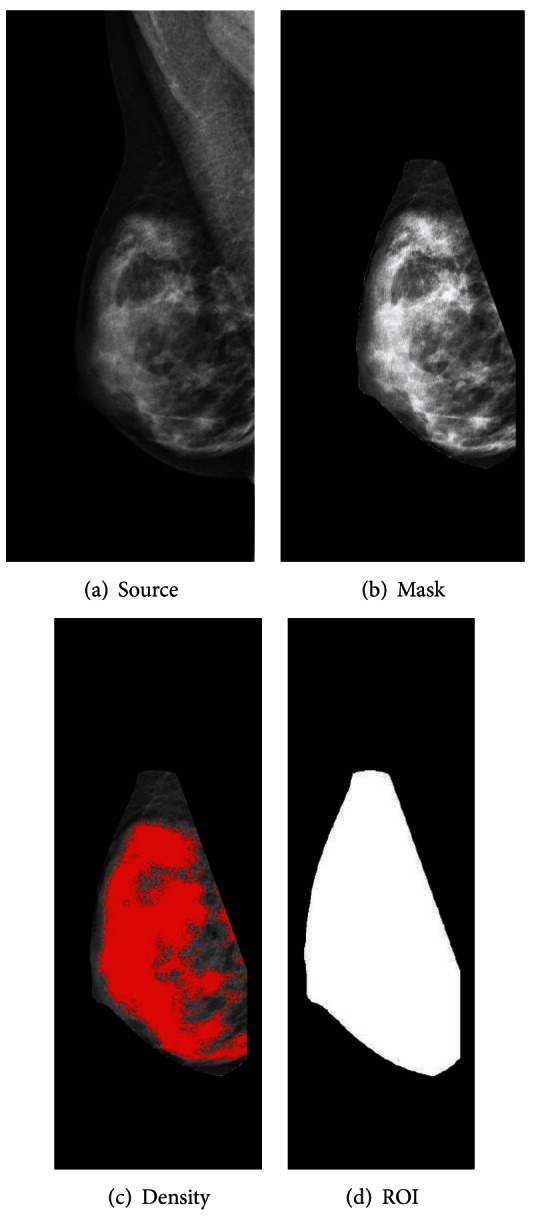
A sequence of processed images generated at various steps of the algorithm for estimating area-based mammographic density: (a) a “For Presentation” mammogram from our sample; (b) the image after a mask has been applied to identify the breast envelope; (c) the area of dense tissue (red pixels); and (d) the region of interest as a binary map of the breast envelope. The algorithm calculates percent breast density as the number of red pixels in Panel (c) divided by the number of white pixels in Panel (d).

 Percent mammographic density was measured by a fully automated research-based algorithm that uses “For Presentation” FFDMs to calculate an area-based measure of density as a percentage on a continuous scale (Figure 1, Panels 1(a) through 1(d)). Using view position and image laterality information from the DICOM header (elements (0018, 5101) and (0020, 0062), resp.) the software creates and applies a mask to identify the breast envelope (region of interest) by removing the pectoral muscle, subcutaneous fat, and overlay text (Panel 1(b)). A variation of the MaxEntropy and Moments thresholding methods is applied to determine a threshold for dense tissue in the breast [22, 23]. The area of the dense tissue (i.e., the number of pixels of dense tissue) is then calculated (Panel 1(c)), as is the area of the region of interest (i.e., the number of pixels in the breast area, Panel 1(d)), and the final density estimate is calculated as the ratio of dense tissue area to the region of interest. In this manner, the software uniquely generates a reproducible, fully automated, area-based estimate of mammographic density using “For Presentation” FFDM images.

To evaluate the agreement between the algorithm and an expert mammographer, percent mammographic density was visually assessed by five radiologists in 5% density increments (0%, 5%,…, 95%, 100%) using five megapixel Barco Screens supported by the Syngo MammoReport Software (VB24D, Siemens AS). Visual assessments were performed by two senior mammographers, one junior mammographer, one senior resident, and one fellow. This 21-point scale was used as a proxy for a continuous measure.

### 2.2. Statistical Analysis

 To quantify the reliability of estimates performed by the radiologists' visual assessments, Intraclass Correlation Coefficients (ICCs) were used to measure interobserver agreement. Although the interpretation of ICCs can vary depending on the context, the ICC is equivalent to a quadratically weighted Kappa, and a widely referenced scale for interpretation of Kappa values can be used as a general guide [24, 25]. Specifically, ICC values of 0.00–0.20, 0.21–0.40, 0.41–0.60, 0.61–0.80, and 0.81–1.00 were used to indicate poor, fair, moderate, substantial, and excellent to perfect agreement, respectively.

It has repeatedly been shown that radiologists' visual assessments of mammographic density are associated with breast cancer risk [1, 3, 4, 10, 26]. As such, the median of the visual assessments performed by the five participating radiologists was considered to be the reference standard for this analysis. The algorithm was considered promising in informing clinical practice if the agreement between the algorithm and the reference standard fell within the 95% CI of the ICC of the radiologists.

The ICC was used to quantify the level of agreement between the algorithm and the reference standard, and a scatterplot was used to demonstrate the relationship between the two. A Bland-Altman difference plot was used to analyze the agreement between the algorithm and the reference standard and to quantify the amount and direction of bias as well as the upper and lower limits of agreement (bias ±1.96*σ* of the difference) [27]. Lastly, a box-and-whisker plot was used to visualize the results in terms of the BI-RADS density lexicon (0–24%, 25–49%, 50–74%, and 75–100%) [7].

## 3. Results

 Five radiologists visually assessed 138 images to estimate mammographic density, and the algorithm was applied to those same 138 images to generate a fully automated density assessment for each of the images.

The radiologists' visual assessments were in excellent agreement with an ICC = 0.884, 95% CI (0.854, 0.910). The algorithm demonstrated excellent agreement with the reference standard with an ICC = 0.862, which fell within the 95% CI of the agreement between the radiologists' visual assessments. The algorithm is validated well on an external set of 261 mammograms, ICC = 0.841.

The Pearson correlation coefficient between the algorithm and the reference standard assessments was *ρ* = 0.889.

**Figure 2:**
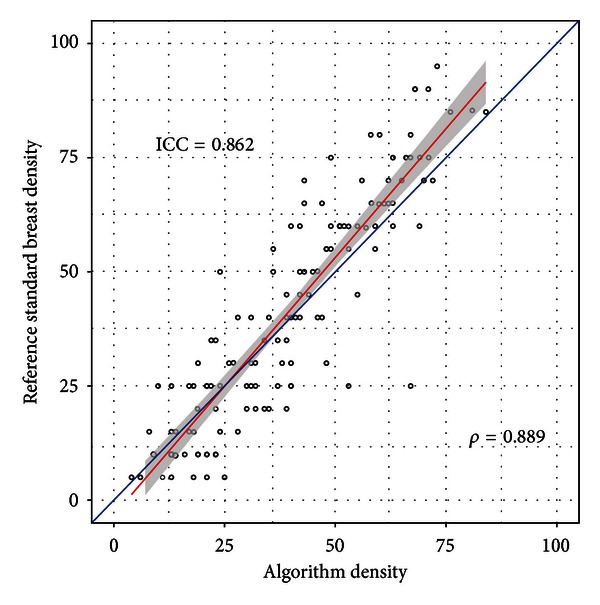
Scatter plot showing the relationship between the mammographic density estimates produced by the algorithm (*x*-axis) and the reference standard (*y*-axis). The blue line indicates perfect agreement between the algorithm and the reference standard, in which case all points would fall exactly on the line of agreement. The red line is the line of the best fit determined by linear least squares regression analysis and shows that the algorithm tends to slightly overestimate density compared to the reference standard for lower densities and slightly underestimate density compared to the reference standard for higher densities.

**Figure 3:**
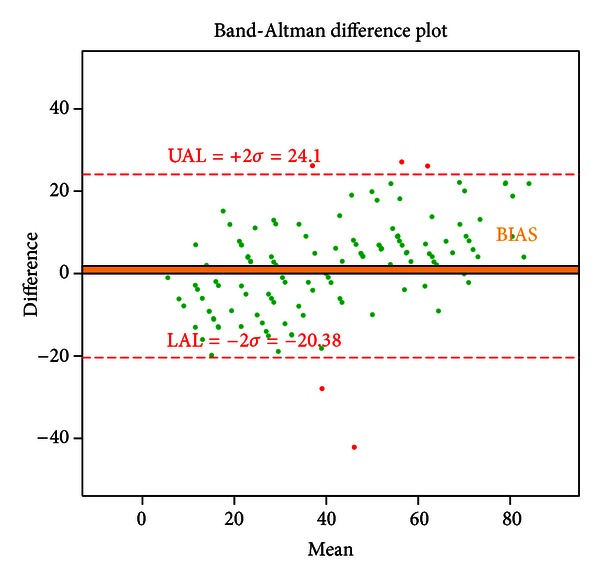
Bland-Altman difference plot showing agreement between the algorithm and the reference standard measures of mammographic density. The difference refers to the reference standard minus the algorithm assessment. The absolute values of the upper and lower agreement limits are <25%, which is the span of a single category in the 4-level BI-RADS density classification scheme. A bias of +1.86%, as indicated by the orange band above the horizontal zero difference line, shows that the reference standard density is on average only slightly higher than the density generated by the algorithm.

The algorithm slightly overestimated low densities and underestimated high densities compared to the reference standard (Figure 2). Overall, there was a small, positive bias in the reference standard assessments compared to the algorithm assessments, as measured by the mean difference between the reference standard and the algorithm assessments (bias = 1.86%) (Figure 3). Additionally, the upper and lower agreement levels were both less than 25%, and thus approximately 95% of the data classified by the algorithm was within one BI-RADS category of the reference standard classification (Figure 3).

**Figure 4:**
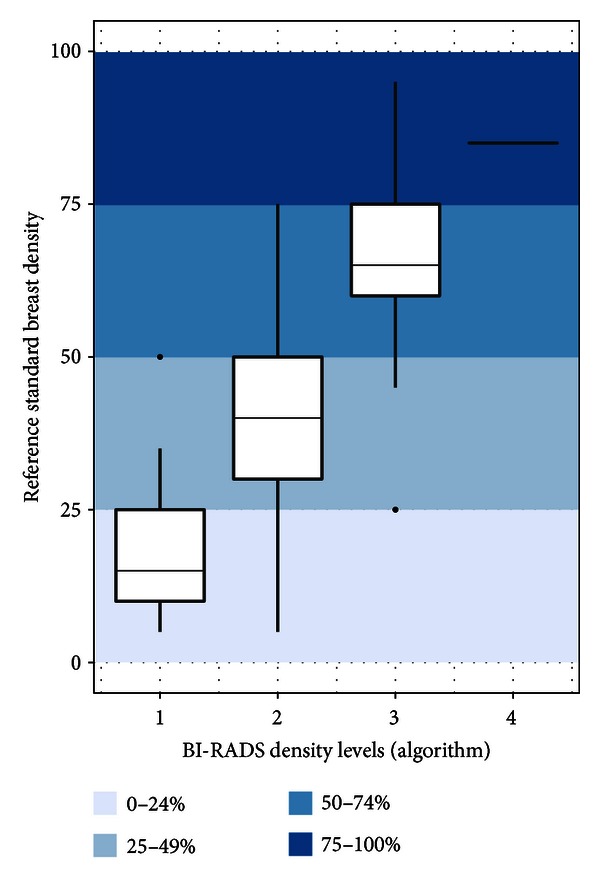
Box-and-whisker plot displaying the distribution of reference standard mammographic density assessments falling into the algorithm-derived classifications designated by the standard 4-level BI-RADS density lexicon. Ideally, each of the boxes and their whiskers should be entirely contained in their respective BI-RADS levels. The reference standard mammographic density assessments in the lowest and the highest BI-RADS levels are well classified, while the middle two levels overlap in both directions into adjacent BI-RADS levels.

When the algorithm and reference standard estimates were classified using the BI-RADS density lexicon, the box-and-whisker plots showed good agreement within categories (Figure 4). Each box was contained in the accordant colour bar, and, as expected from the Bland-Altman difference plot, the tails on the graphs did not exceed the adjacent BI-RADS categories.

## 4. Discussion

 The algorithm demonstrates excellent agreement with radiologists' visual assessments of mammographic density. Critically, the observed magnitude of this agreement falls within the 95% CI of agreement observed between radiologists. This algorithm is unique in that it generates fully automated mammographic density measurements that can be straightforwardly compared with visually determined radiologists' estimates, which are well accepted as being associated with breast cancer risk.

The sole use of the Pearson correlation coefficient (*ρ*) provides a one-dimensional and overinflated impression of the level of agreement.

The statistical evaluation presented in this paper used ICCs and Bland-Altman, scatter, and box plots to quantify agreement and bias in breast density assessment between a fully automated algorithm and radiologists' assessments. This multifaceted methodology can be employed to comprehensively evaluate the performance of any breast density measurement algorithm and provides an alternative to the often reported Pearson correlation coefficient and percent agreement statistics which do not consider random chance agreement and cannot quantify bias between different raters. 

As breast density legislation gains momentum in the USA and mammography providers are required to disclose breast density in the lay report, there will be an increasing need for automated solutions that provide reliable and accurate measurements of breast density. A woman's breast density will be used to determine her optimal followup, and thus the performance of these algorithms must be evaluated using robust statistical methodologies.

## 5. Conclusion

 Further work is needed to extend the applicability of the breast density algorithm to FFDMs from other manufacturers as each manufacturer has their own proprietary image processing algorithms that generate “For Presentation” images. Additionally, as radiologists use both mediolateral and craniocaudal views to assess breast density in a clinical setting, the present algorithm must also be extended to accommodate the analysis of craniocaudal views.

The present algorithm is an effective research tool and shows promise in its ability to provide automated mammographic density measurements that can be used to inform clinical practice. The Pearson correlation coefficient (*ρ*) provides an inadequate, inflated, and overoptimistic measure of the level of agreement. The statistical methods employed provide a comprehensive evaluation of the level of agreement between the algorithm and the reference standard and confirm that the algorithm has an excellent level of agreement with the reference standard. Agreement between raters can only be adequately assessed using multiple statistical methods.
